# Characteristics of Serrated Adenomas in Non-Hispanic Whites and African Americans Undergoing Screening Colonoscopy

**DOI:** 10.7759/cureus.16200

**Published:** 2021-07-05

**Authors:** Lauren Stemboroski, Joshua Samuel, Ahmad Alkaddour, Nicholas Agresti, Ena Gupta, Carlos Palacio, Juan Carlos Munoz, Amie Deutch, John Erikson L Yap, Kenneth J Vega

**Affiliations:** 1 Gastroenterology, University of Florida - Jacksonville College of Medicine (COM), Jacksonville, USA; 2 Internal Medicine, University of Florida - Jacksonville College of Medicine (COM), Jacksonville, USA; 3 Gastroenterology and Hepatology, Augusta University Medical College of Georgia, Augusta, USA; 4 Internal Medicine, University of Florida, Jacksonville, USA

**Keywords:** sessile serrated adenoma, colon cancer and colon polyps, screening colonoscopy, gastroenterology and endoscopy, ethnic disparities

## Abstract

Background and aim

Adenomatous polyps are precursor lesions for colorectal cancer (CRC). Serrated adenomas/polyps are considered a risk factor for the development of proximal and interval CRC. African-Americans are at higher risk for right-sided CRC. Minimal data evaluating serrated adenoma characteristics by race/ethnicity on initial screening colonoscopy (SC) exist. The aim of this investigation was to compare the characteristics of serrated adenomas found in non-Hispanic whites (nHw) and African-Americans (AA) undergoing initial SC.

Methods

The University of Florida-Jacksonville endoscopy database was searched for all SC performed between January 2000 and December 2014. Inclusion criteria were nHw or AA race/ethnicity and histologically proven serrated adenoma found at SC. Data were collected for all included age at SC, sex, number, location, and size of serrated adenomas found.

Results

A total of 8693 individuals (nHw - 4199 and AA - 4494) underwent SC between January 2000 and December 2014. Serrated adenomas were found in 479 individuals (nHw, n=294; AA, n=185), and AA were significantly less likely than nHw to have serrated adenomas on SC (AA 4.1% vs nHw 7%; p< 0.0001). No difference was observed in mean age, location, or size between nHw and AA with serrated adenomas.

Conclusions

Serrated adenomas are more frequent in nHw compared to AA at initial SC. No difference was seen in size or location of serrated adenomas, as well as patient age, between AA and nHw. A study of genetic factors predisposing to serrated adenoma formation and the impact of socioeconomic disparities should be performed across ethnic groups to understand this difference.

## Introduction

Colorectal adenomatous polyps are well-recognized as precursor lesions of colorectal cancer (CRC). The histologic features, size, and number of polyps are predictive factors of CRC and determine future screening surveillance [1]. Hyperplastic and serrated adenomas were initially thought to have little malignant potential. Tubular, tubulovillous, and villous adenomas were considered the predecessor of sporadic CRC [2-3]. However, serrated adenomas have recently been identified as an additional CRC precursor through an alternative pathway and are now considered a target for colonoscopy surveillance, potentially accounting for 15-20% of CRC [2,4]. Serrated adenomas are defined as hyperplastic polyps with a “sawtooth” pattern histologically, and recent investigations have revealed that serrated lesions are precursors to CRC due to distinct genetic mutations such as CPG island methylation and BRAF mutations [4-5]. Serrated adenomas that are large (>1 cm) and proximal have demonstrated a strong association with advanced neoplasia [1,4-5]. Age, male sex, and the number of small adenomas are also predictors of advanced neoplasia and CRC [6].

Colorectal cancer is the third most common cause of cancer in the United States, and the second leading cause of cancer-related death, with racial and ethnic disparities affecting incidence and mortality rate [7-8]. African American (AA) patients have worse stage-specific colorectal cancer survival rates than whites [9]. Recent studies report CRC is affecting certain racial groups at an earlier age than others, prompting changes in the timing of CRC screening based on this factor [7]. In addition, colonic adenomas are more frequent in the AA and Hispanic American (HA) population [1,10]. However ethnic variation in serrated adenoma endoscopic prevalence has not been well-understood. One small study in low-income and uninsured patients observed a reduced risk of serrated polyps in AA as compared to nHw [11]. Furthermore, AA are at increased risk of any conventional right-sided or proximal adenomas [1,11-12]. However, data regarding the frequency and location of serrated polyps is also limited [11].

Minimal data exist from community-based hospitals, with diverse populations evaluating serrated adenoma prevalence by race/ethnicity on initial screening colonoscopy [13]. Current guidelines establishing adenoma detection rates for performance quality were not based on large, diverse populations in community-based hospitals and consisted mostly of nHw populations [8,13]. The aim of this investigation was to compare the characteristics of serrated adenomas found between non-Hispanic whites (nHw) and AA undergoing initial screening colonoscopy at a southern United States safety-net hospital.

The abstract of this article was previously published in the American Journal of Gastroenterology; volume 110; p S585.

## Materials and methods

Patient selection and data collection

The University of Florida-Jacksonville endoscopy database was retrospectively searched for all screening colonoscopies performed between January 1, 2000, and December 31, 2014. Any patient having a colonoscopy with an indication of colon cancer screening, nHw or AA ethnicity, and found to have histologically proven serrated adenomas were eligible for the investigation. Exclusion criteria were individuals of other race/ethnic groups or undisclosed ethnicity and those undergoing colonoscopy for any other indication besides screening. Data collected from the University of Florida-Jacksonville endoscopy database and electronic medical record included age, gender, nHw or AA race, as well as number, location, and size of serrated adenomas found. The Institutional Review Board of the University of Florida at Jacksonville approved the study; IRB approval number 2015-J-0011.

Statistical analysis

Continuous variables were compared as means + standard deviation, prevalence data were recorded as a number and percent of the total group. The student’s t-test or chi-squared test was used for comparison between African Americans and non-Hispanic whites as appropriate. Differences between ethnic groups were considered significant if p <0.05. Data analyses were performed using Stata (version 12.1, Stat Corp, College Station, Texas). Logistic regression analysis was performed using SPSS version 21 (IBM Corp., Armonk, NY) to the model probability of right vs. left-sided serrated adenomas according to gender, race, the number of polyps, the average size of polyps, and age among the 451 cases of serrated adenoma identified.

## Results

A total of 12601 individuals underwent a screening colonoscopy between January 1, 2000, and December 31, 2014. Of those, 8693 individuals having screening colonoscopy were either AA or nHw ethnicity. Overall, the average age was 59.35 (+10.32) years and 44.8% were males. The demographic data of the overall groups by ethnicity is presented in Table 1. A total of 4199 subjects were nHw (48.3%) and 4494 were AA (51.7%). AA undergoing screening colonoscopy were significantly older than nHw (AA, 59.9 + 10.16 years vs nHw, 58.7 + 10.32 years, p <0.001). A higher percentage of males were present in the nHw group compared to AA (nHw 49.5%, n=2078 vs AA 40.4%, n=1817, p <0.001).

**Table 1:** Demographic information by ethnicity SD = Standard Deviation

	African -American	Non-Hispanic whites	Total	p-value
Number (%)	4494 (51.7)	4199 (48.3)	8693	
Age (mean + SD)	59.9 ± 10.16	58.7 ± 10.32	59.35 ± 10.32	<0.001
Male, number (%)	1817 (40.4)	2078 (49.5)	3895 (44.8)	<0.001

Serrated adenomas were found in 479 (5.5% of total) individuals undergoing screening colonoscopy during the investigation period and comprise the study group (Table 2). Serrated adenomas were present in 185 AA (38.6%) and 294 nHw (61.4%). In the cohort of individuals with serrated adenomas, the mean age was similar between AA and nHw (AA 58.9 + 8.8 years vs nHw 59.6 + 8.4 years). However, nHw had a higher percentage of males compared to AA (nHw 51.9%, n=148 vs AA 42.4%, n=76, p-value 0.044). AA were less likely than nHw to have serrated adenomas on screening colonoscopy (AA 4.1% vs nHw 7%; p< 0.0001). The size of serrated adenomas found did not vary between groups (nHw 6.5 mm ± 4.1, AA 6.1 mm ± 4.5, p= 0.175). The logistic regression analysis modeling probability of right vs. left-sided lesions among patients with serrated adenomas demonstrated that average polyp size was associated with increased probability of right-sided lesions for both groups. There was an 11% increase in the association of a right-sided lesion for every increase in 1 mm polyp size (p<.001). For every increase in age by one year, there was an increased risk of 3.9% for right-sided SAs (p=.005). Of the 479 serrated adenoma patients, 347 (72.4%) had only distal colon serrated adenomas, 109 patients (22.7%) had only proximal colon serrated adenomas, and 28 patients (5.8%) had them on both sides. No difference in serrated adenoma location was seen between the two ethnic groups (74.1 % distal in AA, 71.4% distal in nHw, p-value = 0.91). Logistic regression analysis demonstrated no significant association in the probability of right vs. left-sided lesions according to gender, race, or number of polyps.

**Table 2:** Characteristics of patients with serrated adenomas NS = Not Significant SD = Standard Deviation

	African American (N=4494)	Non-Hispanic whites (N=4199)	p-value
Serrated adenoma (N, %)	185 (4.1)	294 (7)	<0.0001
Age (years, mean + SD)	58.9 ± 8.8	59.6 ± 8.4	NS
Male (N, %)	76 (42.4)	148 (51.9)	<0.045
Size of serrated adenoma (mm + SD)	6.1 + 4.5	6.5 + 4.1	NS
Distal location of serrated adenoma (%)	74.1	71.4	NS

## Discussion

The present study of serrated adenoma characteristics at screening colonoscopy between African American and non-Hispanic white patients is one of the largest to date directly comparing serrated adenomas between ethnic groups at a center caring for a large proportion of AA in the United States. It was designed to assess if the characteristics of serrated adenomas (size, location, and frequency) found at screening colonoscopy vary by patient race. The results indicate that serrated adenomas are present more frequently in nHw than AA in north Florida but distribution and polyp size were equivalent by race within the colon. 

The serrated pathway is a relatively recent area of investigation which is rapidly progressing; many studies prior to 2010 failed to recognize or comment on serrated polyp presence and specific locations [3]. Haque et al. indicated limited data exists comparing serrated polyps by ethnic group [3]. Current literature reports a high incidence of proximal lesions in AA, which corresponds to a higher risk of CRC [13-14]. Serrated adenomas are found throughout the colon and rectum, with the majority in the recto-sigmoid colon, however, large (higher risk) serrated adenomas are disproportionately represented in the right colon [15]. The current study confirms no significant difference by race for the risk of distal serrated adenomas, which has been reported by other investigators [11,13]. However, one group examining risk factors for serrated polyps found race to have a significant association with decreased risk of right and left-sided serrated lesions, AA and HA had a relative risk of 0.65% and 0.33%, respectively, compared to Caucasians warranting further investigation [16]. Conflicting data exist on the correlation between *Helicobacter pylori (H. pylori)* and sessile serrated polyps (SSP) prevalence. One study found a direct correlation between H. pylori and SSP prevalence [17] while another study in the US found an inverse relationship between the two [18]. More research on this topic is warranted.

AA have been found to have a higher rate of adenomas as well as more advanced and proximal adenomas than nHw in previous studies, which is considered a potential explanation for increased CRC seen in AA [19]. Lieberman and colleagues found that AA men and women had a higher risk of polyps > 9 mm, a surrogate for advanced neoplasia, and AA men over age 60 were more likely to have proximal polyps > 9 mm in size [20]. Another group reported a higher frequency of proximal polyps in young AA and HA, however, sessile serrated adenomas were not included, as this was not well-cataloged [1]. Schroy et al. found a higher prevalence of advanced colorectal neoplasia (ACN) in nHw compared to AA, specifically nHw men, which is different than current evidence, and observed a predilection of proximal disease among AAs [21]. They speculate the variance in results between studies may be explained by differential exposure to modifiable risk factors for ACN or differences in the primary endpoint (polyp classification vs size) [21].

This study indicates that nHw having a higher incidence of serrated adenomas than AA and the higher frequency of CRC overall in AA cannot be attributed to the serrated adenoma pathway. Health care utilization among AA may play an important role in CRC development within that group. Investigators revealed no meaningful difference in the yield of colorectal neoplasia by race, yet AA were less likely to undergo diagnostic evaluation after abnormal screening results [22-23]. Another group suggests socioeconomic status is associated with a higher CRC incidence independent of individual-level risk, especially for cancers of the left colon where screening is generally more effective [24]. Ollberding and colleagues examined ethnic differences in CRC risk and found known or suspected risk factors of CRC to account for a moderate proportion of CRC risk and other factors, such as genetics and environmental exposures, to be important contributors [25].

A study of genetic factors predisposing to serrated adenoma formation should be performed across ethnic groups to understand the variation observed in the present investigation. A large study done at Howard University showed MUC6, SEMG1, TRNP1, and FSCN1 expression were statistically higher in SSA/Ps compared to HPs; BRAF V600E was identified in 55.6% of SSA/Ps compared to 12.0% of HPs and SSA/P lesions demonstrated higher MLH1 staining intensity (70.3% showing >2+ staining) in comparison to HPs (42.9%) [26]. However, difficulty in discerning various types of serrated lesions has affected the ability to perform epidemiologic analysis [16]. Sessile serrated adenomas have a flat morphology and indistinct borders, making them difficult to detect [4]. Recent investigations assessing adenoma detection rates among endoscopists have shown wide ranges of proximal serrated polyp detection [4]. Hetzel et al. found that the detection rate of sessile serrated adenomas varied significantly by endoscopist, and pathologic identification also varied significantly from 0.0% to 2.2% and 0.0% to 3.9%, respectively [2]. Maratt et al. found that in addition to established factors, body mass index (BMI), endoscopists’ screening ADR, and years since training were associated with ADR, whereas African-American race and diabetes were inversely associated with SSPDR [27]. Poor reproducibility is likely contributing to varying study results. Some centers did not have any sessile serrated adenomas/polyps reported, reinforcing the importance of pathological identification [4]. Given the wide variation in serrated polyp detection and the pathway to CRC, the development of detection rates for sessile adenomas should be considered.

The strengths of our study include information on the size and location of adenomas and a wide distribution of patients by sex, age, and racial/ethnic groups. More importantly, the patients were average-risk patients undergoing initial screening colonoscopy. The community-based design at a safety net hospital reduces selection bias and more likely reflects a "real-world" assessment of serrated adenomas at screening colonoscopy. Furthermore, our study provides information on serrated adenoma detection rates using histological identification.

Potential limitations include the lack of differences of adenoma distribution by gender, as well as environmental factors, such as smoking and chronic diseases, were not evaluated. The investigation was conducted at a single center, thus our findings may not be generalizable to other regions or healthcare settings. The nHw group had a higher percentage of males compared to AA. With male gender being a risk factor for CRC, this may be a source of bias impacting the nHw group, resulting in more proximal lesions. Also, it is well known that AA, especially AA males, have less trust in their providers and of the health care system, overall resulting in decreased colonoscopy for screening purposes [25]. In addition, females are known to have increased serrated adenoma rates compared to males [28-29]. As the AA group in the present investigation had a higher proportion of females than the nHw group, this could have resulted in a higher serrated adenoma rate in AA. In spite of this finding, nHw were found to have an elevated serrated adenoma rate compared to AA, providing further confirmation of nHw race as a risk for serrated adenomas. Finally, serrated polyps have several different histologic types: hyperplastic, sessile, traditional, or mixed; our study did not specify which type of serrated polyp was found. Of note, traditional serrated adenomas are commonly found in the distal colon, which could have resulted in more distal adenomas [6].

## Conclusions

In conclusion, our study indicates the characteristics of serrated adenomas found at screening colonoscopy vary between AA and nHw with regard to frequency only, with nHw more likely to have such lesions at screening colonoscopy. Interestingly, no difference was discovered in the location or size of serrated adenomas found at screening colonoscopy between AA and nHw. In addition, our findings suggest that nHw may be at a higher risk of colon cancer development via the serrated adenoma microsatellite precursor pathway in comparison to AA. Furthermore, prospective investigation of these patients is needed to determine specific serrated adenoma screening and surveillance colonoscopy recommendations. Continued investigation is required to determine identifiable risk factors, such as genetic, clinical, environmental, and socioeconomic factors, for proximal serrated adenoma formation and progression to colon cancer based on the disparity observed.
